# Integration of Digital Denture Technology in the Prosthodontic Management of Pediatric Osteopetrosis: A Case Report

**DOI:** 10.1155/crid/2537509

**Published:** 2026-04-20

**Authors:** Athina Niakou, Savvas Kamalakidis, Athanasios Stratos, Konstantinos Arapostathis, Eleni Kotsiomiti

**Affiliations:** ^1^ Department of Prosthodontics, Faculty of Dentistry, School of Health Sciences, Aristotle University of Thessaloniki, Thessaloniki, Greece, auth.gr; ^2^ Division of Postgraduate Prosthodontics, School of Dental Medicine, Tufts University, Boston, Massachusetts, USA, tufts.edu; ^3^ Department of Pediatric Dentistry, Faculty of Dentistry, School of Health Sciences, Aristotle University of Thessaloniki, Thessaloniki, Greece, auth.gr

**Keywords:** growth-adaptable prosthesis, osteopetrosis, pediatric prosthodontics, removable partial denture

## Abstract

Osteopetrosis is a rare hereditary disorder characterized by impaired osteoclastic bone resorption, resulting in generalized skeletal sclerosis, increased bone fragility, and a heightened risk of complications such as osteomyelitis. Prosthodontic rehabilitation in pediatric patients is particularly challenging due to ongoing craniofacial growth, incomplete dental development, and the need to avoid invasive procedures. A pediatric patient with osteopetrosis presented with mandibular Kennedy Class I, Modification I partial edentulism, misalignment of the maxillary teeth, and dentition at varying stages of development. A conservative removable prosthodontic approach was selected. Following tissue conditioning, a functional “washout” impression using a soft liner was performed to record the supporting tissues while minimizing pressure. To permit flexibility and future modification during growth, a removable partial denture (RPD) without a metal framework was fabricated. The impression surface was digitized using an intraoral scanner (TRIOS, 3Shape, Copenhagen, Denmark). The scan data guided computer‐aided design and milling of a new RPD from a monolithic polymethyl methacrylate (PMMA) disk (Ivotion, Ivoclar Vivadent AG, Schaan, Liechtenstein). The tissue‐borne prosthesis was extended to maximize support and contoured around the canines to provide encirclement and reciprocation, enhancing stability and retention while reducing stresses on abutment teeth. The digitally fabricated denture demonstrated an accurate intaglio fit, minimal intraoral adjustments, and improved esthetics and masticatory function. Its design allowed repeated tissue conditioning, incremental adaptation, and modifications to accommodate craniofacial growth. All digital scans were archived, enabling longitudinal superimposition to monitor growth‐related changes and facilitate early identification of pressure areas or sites at risk for osteomyelitis. Recall appointments were essential for growth adjustments and monitoring. Within the limitations of this case, an acrylic resin RPD without a metal framework represents a functional and adaptable interim solution for pediatric patients with osteopetrosis. The integration of digital workflows supports long‐term follow‐up and early complication detection during growth.

## 1. Introduction

Osteopetrosis is a rare hereditary bone disorder characterized by impaired bone remodeling and increased bone density caused by defective osteoclastic resorption [1, 2]. Initially described in 1904, it is also known as “marble bone disease,” “Albers‐Schönberg disease,” or “osteosclerosis fragilis generalisata” [3–5]. The genetic heterogeneity of the disease results in a broad spectrum of phenotypic expressions, ranging from asymptomatic to severe forms. Osteopetrosis is classified into three clinically distinct forms based on severity [6].

The severe infantile form, inherited in an autosomal recessive pattern, usually presents at birth or within the first few months of life. It is characterized by densely sclerotic bones, frequent fractures, and neurological complications, most notably optic nerve compression leading to visual impairment. Additional features include bone marrow failure, recurrent infections, and a bleeding tendency, as well as early mortality. Without treatment, affected infants rarely survive beyond their first 2 years of life [7, 8].

The intermediate form follows either an autosomal dominant or autosomal recessive inheritance pattern, presenting milder symptoms while remaining clinically severe. It is associated with brain calcifications and renal tubular acidosis, as a result of mutations in the carbonic anhydrase II (CAII) gene. Further characteristics include intellectual disability, mild skeletal sclerosis, short stature, and an increased risk of fractures [8, 9].

The mild or late‐onset form (adult type) follows an autosomal dominant pattern and exhibits clinical heterogeneity. It is characterized by thickening of the vertebral end plates, pelvis, and skull base, often accompanied by diffuse bone pain, hematologic and neurologic complications, osteomyelitis, and pathological fractures [8–10].

Autosomal recessive osteopetrosis is rare, with an incidence of approximately 1:250,000 births, whereas the autosomal dominant form occurs in about 5:100,000 [11]. In the craniofacial region, dental findings include delayed eruption, malformed or missing teeth, increased caries susceptibility, and high risk of osteomyelitis [12–16].

Pediatric patients with osteopetrosis present challenges for prosthodontic rehabilitation due to altered bone metabolism, abnormal alveolar anatomy, and a higher risk of osteomyelitis. These cases necessitate meticulous treatment planning, careful material selection, and minimally invasive approaches [17]. Multidisciplinary collaboration among pediatric dentists, prosthodontists, and oral surgeons is essential to ensure functional and esthetic outcomes while minimizing complications [12, 14]. Prosthodontic intervention in these patients aims to restore masticatory function, improve facial esthetics, and protect the remaining oral structures and support oral hygiene [18]. Reports on prosthodontic management in pediatric osteopetrosis remain limited, underscoring the need to share clinical experiences to inform evidence‐based practice.

Digital techniques provide practical advantages in pediatric osteopetrosis. Intraoral scanning of a functional impression can capture the soft‐tissue–conditioned intaglio surface without additional chairside trauma. CAD/CAM fabrication enables reproducible adaptation and simplified remakes as the child grows [19, 20]. Archiving sequential scans generates an objective dataset for superimposition across recalls, facilitating growth surveillance and the early detection of pressure‐related changes or sites at risk for osteomyelitis [21].

This case report presents the long‐term prosthodontic management of a growing child with osteopetrosis, addressing both functional and esthetic rehabilitation while managing recurring osteomyelitic complications through a conservative, adaptable prosthetic approach.

## 2. Clinical Report

### 2.1. Dental History and Preprosthetic Procedures

A 7‐year‐old girl was referred in 2016 to the Department of Pediatric Dentistry with a chief complaint of swelling at her right cheek. Her medical history reported transient anemia in early childhood, while her dental history included a dentoalveolar abscess treated with antibiotics 10 days earlier. Clinical examination revealed short stature, mild frontal prominence, and strabismus. Clinical and radiographic examination revealed rampant caries, rotated and lingually inclined mandibular incisors, Grade III tooth mobility, multiple missing permanent teeth with two supernumeraries near the mandibular lateral incisors, and generalized tooth deformities. The family history did not disclose similar findings [12].

The patient was referred to a Pediatric Neurology Clinic for further medical evaluation. During hospitalization, the permanent mandibular right first molar and both the primary mandibular right first and second molars were extracted. Postoperatively, osteonecrosis developed at the extraction site, which progressed to osteomyelitis and was managed with parenteral antibiotics (ceftriaxone and clindamycin). During the same admission, a diagnosis of osteopetrosis was established by the medical team. Further extractions of severely carious primary teeth were performed, and due to the patient’s high caries risk, close follow‐up was required. Recurrent infections and osteonecrotic lesions necessitated multiple surgical interventions, including debridement and extraction of supernumerary and non‐restorable teeth under general anesthesia [12].

Genetic testing in 2017 did not identify any of the previously known genes related to osteopetrosis and high bone density disorders (CA2, CLCN7, LRP5, OSTM1, PLEKHM1, TCIRG1, TNFSF11, TNFRSF11A, ANKH, COL1A1, GJA1, HPGD, MTAP, SLCO2A1, SNX10, SOST, TBXAS1, TGFB1, TNFRSF11B, and TYROBP) [22–26]. Based on clinical, radiographic, and genetic findings, a diagnosis of intermediate form osteopetrosis was established. Deletion/duplication analysis and whole‐exome sequencing were recommended to clarify the diagnosis.

### 2.2. Prosthodontic Treatment

**Figure 1:**
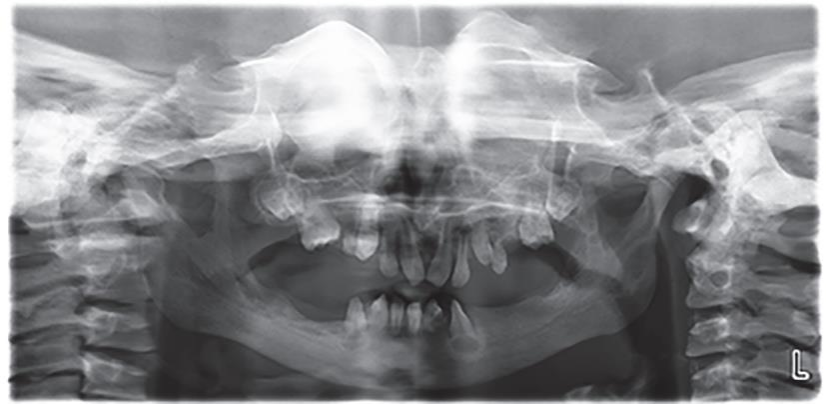
Orthopantomogram showing the initial condition.

The patient was first examined in 2016 following an abscess and diagnosed with osteopetrosis the same year. Preventive and surgical management continued from 2016 to 2021, and prosthodontic rehabilitation was initiated in 2022, with follow‐up continuing until 2025. Between 2017 and 2022, the patient was maintained on a preventive‐focused dental program with frequent recalls. In 2022, she expressed reluctance to smile and difficulties with mastication. However, her primary concern was esthetic rather than functional improvement. Consequently, she was referred to the Department of Prosthodontics by the Department of Pediatric Dentistry. Informed consent for treatment and publication was obtained from the patient’s parents in writing, including permission to use anonymized clinical photographs, before initiating prosthodontic rehabilitation. Clinical and radiographic assessment (Figure 1) demonstrated a young patient in mixed dentition, with Kennedy Class I Modification I partial edentulism in the mandible. Several deciduous teeth remained, while the permanent successors were in various stages of development. The roots of many permanent teeth were still incompletely formed, consistent with the patient’s young age. In the maxilla, the permanent incisors and first molars were erupted, whereas the canines and premolars were unerupted but developing. Notably, the maxillary anterior teeth exhibited rotations and a midline discrepancy. In the mandible, the permanent incisors had erupted, but their roots were still incompletely developed, and all the posterior teeth were missing. Prosthetic rehabilitation with a mandibular removable partial denture (RPD) was planned as a long‐term interim prosthesis to restore esthetics and function, maintain arch integrity, support the developing dentition during craniofacial growth, and prevent further deterioration of the oral structures.

**Figure 2:**
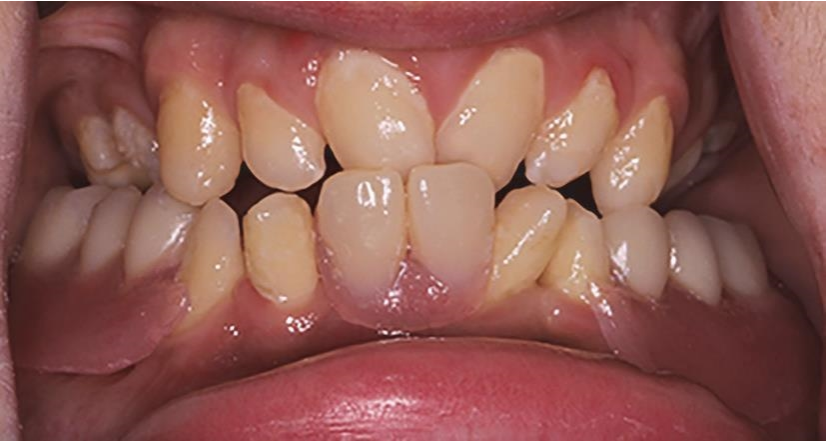
Frontal view of long‐term interim prosthesis.

Stock metal trays were customized to ensure accurate extension of the impressions. Preliminary impressions were made with irreversible hydrocolloid (Cavex Color Change; Cavex Holland BV, Haarlem, The Netherlands) and diagnostic casts were fabricated. The mandibular diagnostic cast was surveyed to establish the most favorable insertion path for the prosthesis. No modifications to the existing teeth were made, and no metal framework was used. The long‐term interim acrylic RPD was planned with 0.02 inch undercuts at the distobuccal surfaces of the mandibular canines to provide mechanical retention via acrylic clasps. Final impressions were made with border‐molded custom trays and a polysulfide elastomeric impression material (Permlastic Polysulfide Rubber Base Impression Material; Kerr Manufacturing Co., Romulus, MI). Interocclusal records were obtained using a light‐activated acrylic resin base (Triad VLC Resin; Dentsply Sirona, York, PA) with a wax occlusal rim, at the patient’s existing vertical dimension and jaw relationship. To enhance support, the denture base was extended to cover the buccal shelf area. At the same time, it was contoured around the canines to achieve guide planes for insertion, embracing, encirclement, and reciprocation. These design features were intended to improve horizontal stability and to minimize the functional stresses transmitted to the abutment teeth. Acrylic denture teeth (SR Orthotyp PE; Ivoclar Vivadent AG, Schaan, Liechtenstein) were arranged in a bilaterally stable cusp‐to‐fossa or cusp‐to‐an acrylic ramp at the lingual side of the denture. This occlusal relationship was achieved in the canine and premolar areas, as the patient is in an Angle’s Class III relationship with minimal ability for excursive movements. A wax try‐in of the acrylic teeth was performed to evaluate esthetics, phonetics, and occlusion. Finally, the mandibular RDP was processed in a heat‐activated acrylic resin (Lucitone 199; Dentsply Sirona, York, PA). The delivery of the prosthesis resulted in improved mastication. It provided satisfactory esthetics and fulfilled the patient’s chief complaint (Figure 2). Follow‐up visits were scheduled at short intervals (1–3 months) to allow close monitoring of the case, given the complexity of the condition and the fragile oral environment. During a recall visit, the mandibular permanent lateral incisors were extracted due to increased mobility, and the RPD was adjusted accordingly. Later, during another recall appointment, the patient presented with a midline fracture at the base of the prosthesis, which was repaired with internal metal wire reinforcement.

**Figure 3:**
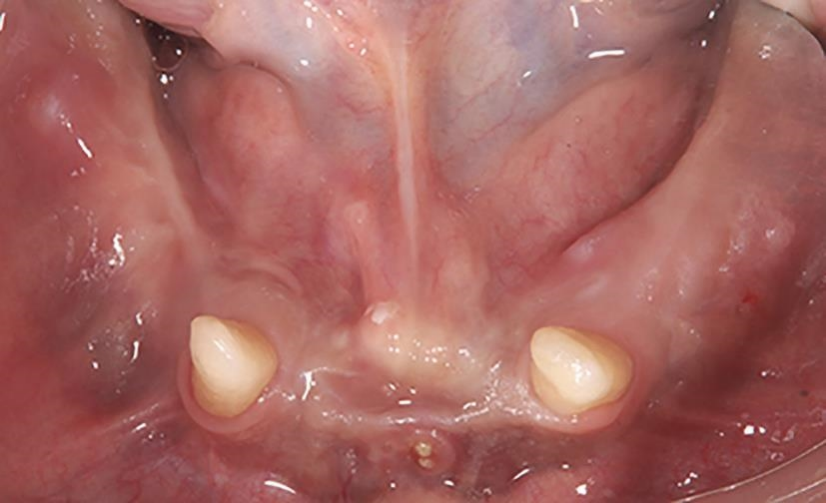
Intraoral view of the mandible and bone trabeculae in the #33–43 area.

**Figure 4:**
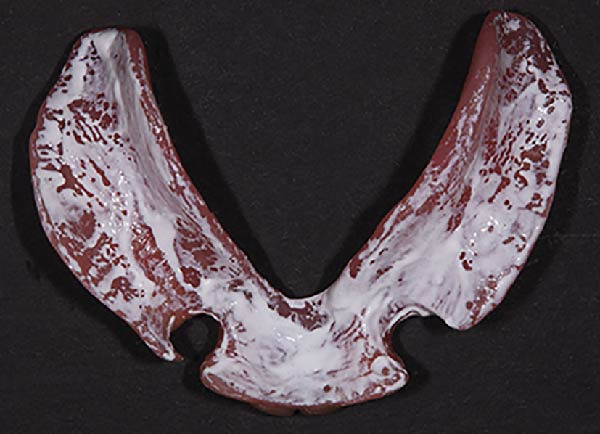
Areas of excessive pressure with pressure‐indicating paste (PIP).

**Figure 5:**
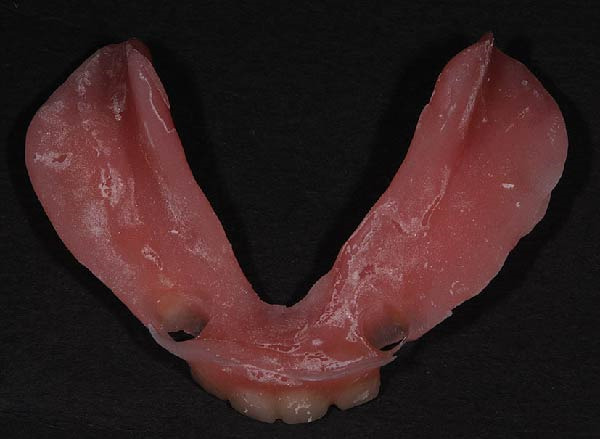
Application of short‐term soft tissue liner.

**Figure 6:**
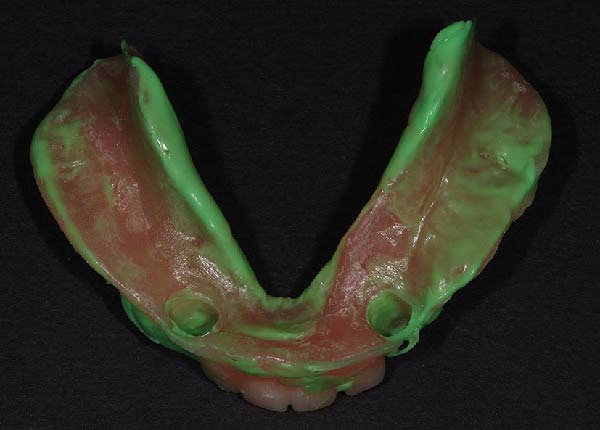
Soft‐liner–based functional impression.

At a subsequent recall in June 2025, the patient reported discomfort in the mandibular intercanine region and reduced retention. Clinical examination revealed exposed bone trabeculae in the mandibular intercanine area and at the posterior regions bilaterally, none of which required surgical intervention (Figure 3). Due to the osteopetrotic nature of the bone, characterized by reduced vascularity and an increased risk of osteonecrosis, a conservative nonsurgical approach was selected, consisting of prosthetic adjustment and tissue conditioning to avoid triggering osteomyelitis. Areas of excessive pressure were first identified using pressure indicating paste (Mizzy PIP; Keystone Industries, Gibbstown, NJ) and selectively relieved (Figure 4). A short‐term tissue conditioner (Viscogel; Dentsply Ltd., Addlestone, Surrey, UK) was applied to the fitting surface of the denture base, after careful reduction of flange borders and relief of the basal area, to improve comfort (Figure 5). The liner was extended onto the external surface of the base and molded through functional muscle movements. The soft tissue liner remained in place for 1 week, after which it was replaced and left in place for 1 day. Afterward, a soft tissue liner‐based functional impression was made using a light‐body addition silicone material (Express Light Body, 3M ESPE, St. Paul, MN; Figure 6).

**Figure 7:**
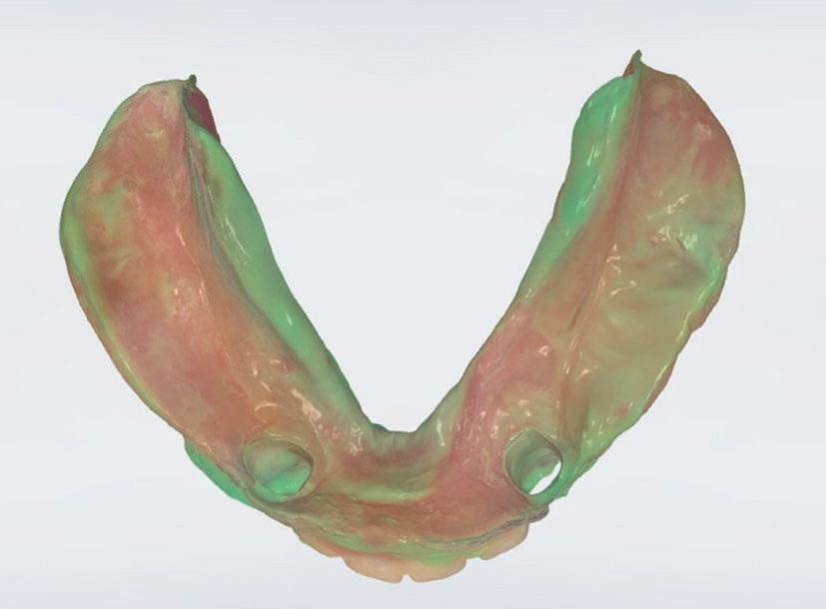
Soft‐liner–based scanned functional impression.

**Figure 8:**
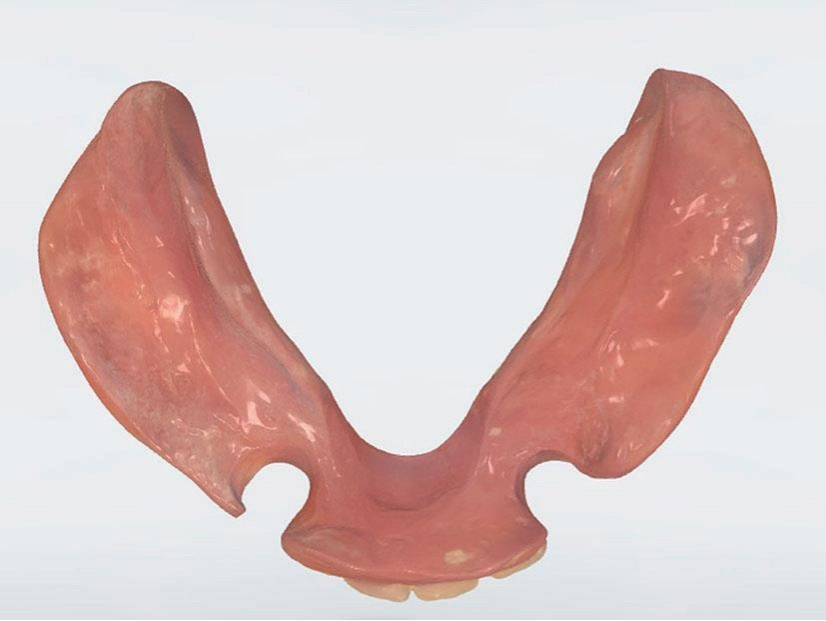
Intaglio surface of designed long‐term interim restoration.

**Figure 9:**
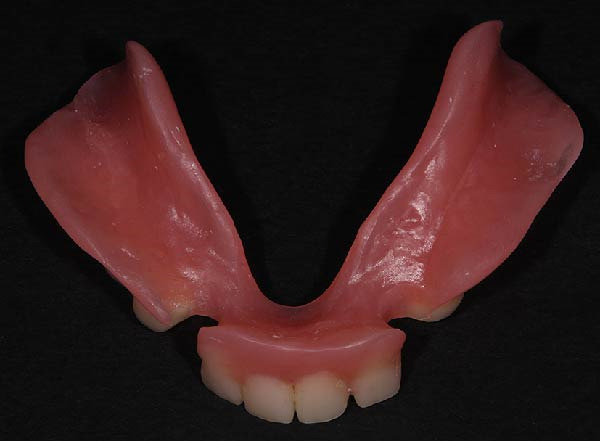
Intaglio surface of long‐term interim restoration.

**Figure 10:**
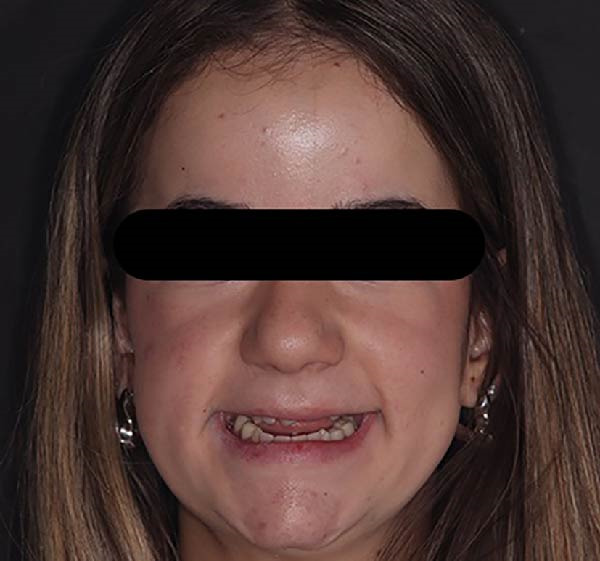
Extraoral view of the long‐term interim prosthesis.

The functional impression was scanned using an intraoral scanner (TRIOS 3; 3Shape, Copenhagen, Denmark) to obtain a digital replica of the intaglio surface and border extensions (Figure 7). This dataset was subsequently used to design (Dental System; 3Shape, Copenhagen, Denmark) and mill (PrograMill7; Ivoclar Vivadent AG, Schaan, Liechtenstein) a new mandibular RPD in monolithic polymethyl methacrylate (PMMA; Ivotion; Ivoclar Vivadent AG, Schaan, Liechtenstein), replicating the analog design while optimizing the tissue adaptation and therefore support. The digitally fabricated prosthesis maintained the exact contours, clasp geometry, and buccal shelf coverage as the conventional one, ensuring comparable retention, horizontal stability, and comfort (Figures 8 and 9). The restoration was delivered to the patient (Figure 10). All digital scans were archived as baseline data for growth monitoring and longitudinal comparison, allowing superimposition of subsequent scans to assess soft‐tissue adaptation, prosthesis fit, and craniofacial growth overtime.

A structured recall and maintenance protocol was established, with visits every 3 months during the first year and semiannual visits thereafter to ensure continuous monitoring and optimal performance of the prosthodontic restoration. The patient and her parents expressed satisfaction with the esthetic and functional results. They mentioned that the prosthesis enhanced the child’s mastication and increased her willingness to smile in public.

## 3. Discussion

Osteopetrosis significantly affects the craniofacial region, posing challenges in dental management [27]. The increased bone density and impaired bone remodeling complicate surgical and prosthetic interventions, while susceptibility to osteomyelitis remains high [2, 28]. In pediatric patients, these challenges are further complicated by the ongoing craniofacial growth [29]. Consequently, prosthodontic treatment must be carefully planned to restore function and esthetics while minimizing biological risks [30].

The prosthodontic rehabilitation of pediatric patients with osteopetrosis is complicated by altered bone metabolism, craniofacial proportions, and dental anomalies [31]. In the present clinical report, the occurrence of mandibular osteomyelitis following extractions underscores the susceptibility of these patients to infection, due to poor vascularity, obliteration of marrow spaces, and compromised wound healing [10, 32]. The observation of exposed bony trabeculae during follow‐up further highlights the chronic risk of secondary infection and the importance of continuous monitoring and early intervention [33]. A balance between aggressive surgical management when indicated and minimally invasive prosthetic procedures is therefore essential to reduce the risk of aggravating osteomyelitic complications [34, 35].

From a prosthodontic perspective, design and maintenance must be adapted to the fragile oral environment of patients with osteopetrosis. The use of soft relining materials should be approached with caution due to their porosity and potential for microbial colonization [36, 37]. In the present case, a temporary soft tissue liner was applied under controlled conditions for a limited time, followed by replacement with heat‐cured acrylic resin to minimize microbial colonization and enhance denture fit and patient comfort. This protocol aimed to prevent local trauma and reduce the risk of osteomyelitis recurrence. In the present case, an acrylic denture without a metal framework was selected. The absence of rigidity and the mild degree of flexibility were advantageous in this context, as they facilitated incremental adaptation and made modification at subsequent recall appointments easier until the young patient’s growth was complete [38].

Digital scanning of the functional impression improved accuracy and documentation. The intraoral scan captured fine surface details of the soft‐tissue liner impression, which were used to digitally design and mill a new denture base with improved precision. The digital dataset also provided a reproducible, noninvasive record of the patient’s intraoral condition, allowing superimposition of subsequent scans using a specialized surface‐matching software program (Geomagic Control X; 3D Systems, Rock Hill, SC, USA), enabling three‐dimensional comparison of changes overtime [23, 39]. This enables early identification of complications such as bone exposure or inflammation, minimizing chairside adjustments and protecting fragile tissues [40]. Moreover, the use of milled PMMA improved surface density and polishability, reducing microbial adhesion and eliminating residual monomer exposure [41]. CAD/CAM technology improved the fabrication accuracy and enabled longitudinal monitoring of the osteopetrosis.

Another consideration is the feasibility of dental implants in patients with osteopetrosis. While implants provide superior functional and esthetic rehabilitation, the sclerotic, poorly vascularized bone environment of osteopetrosis may compromise osseointegration and increase the risk of postoperative infection and osteonecrosis [11]. Dense bone also causes overheating during drilling for implant placement, which can lead to bone necrosis and implant failure. Nonetheless, there are case reports of successful implant‐supported prostheses in carefully selected adult patients [42–44]. However, in pediatric patients, the young age inherently limits the feasibility of dental implants or extensive fixed prosthetic solutions, making a RPD the treatment of choice [12].

Pediatric patients with osteopetrosis often face severe esthetic and functional limitations due to delayed eruption, enamel and dentin anomalies, and structural dental abnormalities [12, 45]. The esthetic zone impairment may cause psychosocial distress, further aggravated by the collapse of unsupported soft tissues [46]. In osteopetrosis, these dental abnormalities are compounded by systemic hypocalcification of bone and teeth, constricted neurovascular canals, and obliterated pulp chambers. Such features increase the risk of caries, tooth loss, and osteomyelitis following extractions, complicating the long‐term oral rehabilitation [13, 45, 47]. Therefore, prosthetic intervention should not only aim to restore function and esthetics but also to preserve oral health and psychological well‐being in a medically compromised child [38, 48].

As a single case report, the findings cannot be applied to all types of osteopetrosis. However, an extended observation period of over 8 years and thorough follow‐up improve the report’s validity and educational value. Additional case series or multicenter studies are necessary to develop standardized prosthodontic protocols for similar medically compromised pediatric patients.

The recall schedule for these patients should be shorter than that for healthy individuals [13, 49]. In the present clinical report, close follow‐up intervals of 1–3 months enabled the early detection of prosthesis‐related pressure areas and bone exposure, allowing timely prosthetic adjustments and preventive interventions. In this context, recall appointments represent the core of treatment rather than routine follow‐up, as they provide essential opportunities for continuous modifications, whether to relieve discomfort from bone or soft tissue inflammation or to adapt the RPD to the mucosa and abutment teeth to maintain support, retention, and stability. Therefore, such a protocol is crucial for the early identification and management of disease‐related complications, as well as for modifying the RPD to accommodate the patient’s skeletal growth.

## 4. Conclusion

Prosthodontic rehabilitation in pediatric patients with osteopetrosis presents unique challenges due to altered metabolism, susceptibility to osteomyelitis, and complex dental anomalies. This case demonstrates that a prosthetically driven, multidisciplinary approach can provide both functional and esthetic improvements in medically compromised patients. Continuous follow‐ups and preventive dentistry are essential for monitoring prosthesis performance, maintaining oral health, and facilitating the early detection and management of osteopetrosis‐related complications. Overall, the long‐term course of the present case underlines the need for individualized treatment planning, careful prosthesis maintenance, cautious surgical management, gentle clinical handling, and adaptable modification of the prosthetic design in response to growth changes and disease progression. The incorporation of a digital workflow added precision, reproducibility, and a new capacity for growth documentation. The archived scan data provide an invaluable digital baseline for serial comparison, enabling quantitative monitoring of craniofacial development and early recognition of tissue complications.

This case report was conducted in accordance with the Declaration of Helsinki. According to institutional guidelines, ethical approval was not required for a single anonymized case report. Written informed consent for treatment and publication, including clinical images, was obtained from the patient’s parents.

## Funding

This study did not receive specific funding. The publication of this article in OA mode was financially supported by HEAL–Link.

## Disclosure

All authors have read and approved the final version of the manuscript. Athanasios Stratos, as the corresponding author and manuscript guarantor, had full access to all of the data in this study and takes complete responsibility for the integrity of the data and the accuracy of the data analysis.

## Consent

Consent statement for treatment and publication was obtained from the patient’s parents in writing, including permission to use anonymized clinical photographs.

## Conflicts of Interest

The authors declare no conflicts of interest.

## Data Availability

All data supporting the findings of this case report, including clinical images and digital scans, are included within the manuscript and its supporting information. No additional data are available.
